# The postmastectomy pain syndrome: an epidemiological study on the prevalence of chronic pain after surgery for breast cancer

**DOI:** 10.1038/sj.bjc.6604534

**Published:** 2008-08-05

**Authors:** O J Vilholm, S Cold, L Rasmussen, S H Sindrup

**Affiliations:** 1 Department of Neurology, Odense University Hospital, Sdr. Boulevard 29, Odense 5000, Denmark; 2 Department of Oncology, Odense University Hospital, Sdr. Boulevard 29, Odense 5000, Denmark; 3 Department of Surgery, Odense University Hospital, Sdr. Boulevard 29, Odense 5000, Denmark

**Keywords:** breast cancer, neuropathic pain, PMPS, postoperative pain, intercostobrachial neuropathy

## Abstract

The prevalence of the postmastectomy pain syndrome (PMPS) and its clinical characteristics was assessed in a group of patients who had undergone surgery for breast cancer at the Department of Surgery, Odense University Hospital, within the period of 1 May 2003 to 30 April 2004. The study included 258 patients and a reference group of 774 women. A questionnaire was mailed to the patients 1½ year after surgery and to the women in the reference group. The PMPS was defined as pain located in the area of the surgery or ipsilateral arm, present at least 4 days per week and with an average intensity of at least 3 on a numeric rating scale from 0 to 10. The prevalence of PMPS was found to be 23.9%. The odds ratio of developing PMPS was 2.88 (95% confidence interval 1.84–4.51). Significant risk factors were as follows: having undergone breast surgery earlier (OR 8.12), tumour located in the upper lateral quarter (OR 6.48) and young age (OR 1.04). This study shows that, although recent advances in the diagnostic and surgical procedures have reduced the frequency of the more invasive surgical procedures, there still is a considerable risk of developing PMPS after treatment of breast cancer.

Within the last decade, it has been increasingly accepted that chronic pain can be a complication to surgery (Perkins and Kehlet, 2000; Macrae, 2001). Chronic pain has been reported after a range of different types of surgery, such as thoracotomy, inguinal hernia repair and hysterectomy.

Similarly, the prevalence of chronic pain after surgery for breast cancer, the postmastectomy pain syndrome (PMPS), has been known to develop in 20–68% of patients (Ivens *et al*, 1992; Maunsell *et al*, 1993; Stevens *et al*, 1995; Tasmuth *et al*, 1995; Wallace *et al*, 1996; Carpenter *et al*, 1998; Smith *et al*, 1999; Kuehn *et al*, 2000; Peintinger *et al*, 2003).

The description, the postmastectomy pain syndrome, is somewhat misleading, as the syndrome also includes chronic pain after breast conserving surgery. The exact mechanism of development is not known, but it is probably a neuropathic pain condition, which is because of damage to the nerves in the axilla and/or the chest wall during surgery. On this basis, an alternative term, intercostobrachial neuralgia, has been proposed (Jung *et al*, 2003). PMPS can develop shortly after surgery or up to several months after surgery and can persist for years. Other causes for chronic pain after treatment for breast cancer include intercostal neuromas (Rosso *et al*, 2000; Wong, 2001; Kretschmer *et al*, 2002).

The prevalence of PMPS has been shown to be higher after lumpectomy than after mastectomy (Tasmuth *et al*, 1996, 1997). The pain is often located in the axilla, the shoulder, the arm or the chest wall. PMPS is often described as a typical neuropathic pain consisting of burning pain, shooting pain, pain evoked by pressure and deep blunt pain. Like other neuropathic pain conditions, the treatment is often difficult. A study from 1994 and a recent study suggest that the prognosis of PMPS is better than expected, with a decline in prevalence over years (de Vries *et al*, 1994; Macdonald *et al*, 2005).

Previous studies have identified following risk factors: young age (Tasmuth *et al*, 1995; Smith *et al*, 1999; Poleshuck *et al*, 2006), sectioning of the intercostobrachial nerve (Abdullah *et al*, 1998; Torresan *et al*, 2003) and axillary dissection (Vecht, 1990; Maunsell *et al*, 1993; Hack *et al*, 1999; Kakuda *et al*, 1999; Johansen *et al*, 2000). Dissection of axillary lymph nodes has been shown to be a critical component in the aetiology of chronic pain after surgery for breast cancer. The frequency of this procedure has been reduced over the past few years, due to the introduction of the sentinel node biopsy (Shons and Cox, 2001). The morbidity of this procedure has been shown to be less than standard axillary treatment (Kakuda *et al*, 1999; Schijven *et al*, 2003; Mansel *et al*, 2006). Thus, the prevalence of PMPS may have decreased (Miguel *et al*, 2001). This study estimates the current prevalence of PMPS and identifies risk factors.

## Materials and methods

A postal survey concerning pain after surgery for breast cancer was undertaken in the County of Funen, Denmark. Invitations to participate, along with questionnaires, were mailed to a group of breast cancer patients, 1½ year after their surgery and to a group of reference subjects. This time point was chosen to be sure to enrol all chronic pain complaints and to avoid acute and transient postoperative pain complaints. The inclusion criterion was as follows: women who had undergone surgery for breast cancer with either mastectomy or lumpectomy, performed at the Department of Surgery, Odense University Hospital within the period from 1 May 2003 to 30 April 2004. The exclusion criteria were as follows: (a) living outside the County of Funen, (b) having undergone thoracotomy, (c) severe angina pectoris. Women with reoccurrence of breast cancer and women who had undergone breast surgery earlier for cancer or cosmetic reasons were included in the study.

The reference group was randomly selected from the same general population as the breast cancer patients. For each breast cancer patient, three reference subjects, who had not undergone surgery for breast cancer, were selected as controls. The reference group was established from the Danish Civil Registry and stratified to the group of breast cancer patients on age and postal code.

A questionnaire was developed (available on request from the authors). Questions about pain focused on present pain and pain experienced in the week preceding the assessment. There were also questions about the location, the intensity and the character of the pain as well as the impact of pain on daily living, the consumption of analgesics and demographic questions. The questionnaires were tested for comments on colleagues and controls. Non-responders received a reminder 1 month after the first mailing. If needed, participants were contacted by telephone for clarifying responses. The Ethical Committee of Vejle and Funen County (VF 20040191) and the Danish Data Protection Agency (J.nr. 2004-41-4514) approved the study, which was performed according to the Declaration of Helsinki.

Information about the surgery and the postoperative treatment for the group of breast cancer patients was obtained from the DBCG registry (Danish Breast Cancer Cooperative Group), the Department of Surgery and the Department of Oncology, Odense University Hospital. Comparisons regarding demographic characteristics were conducted using *χ*^2^ or Fisher's exact test for categorical data, and non-parametric Mann–Whitney *U*-test was used for non-categorical data. Univariate logistic regression analysis was applied for testing the possible risk factors. Afterwards, multiple regression analysis was applied for the analysis of possible risk factors, and *a priori* the following variables were included: age, chemotherapy, radiation therapy, mastectomy, tumour located in the upper lateral quarter. *Post hoc*, it was decided to add the following variables for the multivariate analysis: having undergone breast surgery earlier, smoking and axillary dissection. These variables were significant or near-significant risk factors in the univariate analysis. Age and tumour size were non-categorical variables. All other variables in the multiple regression analysis were categorical variables. Concerning pain and pain characteristics, odds ratios with 95% confidence interval were calculated. Significance was defined as *P*<0.05. All analysis was performed with the statistical program STATA version 8. Individuals with missing information from the questionnaire were excluded from the specific analysis.

For the group of breast cancer patients, the PMPS was defined as pain located in the area of the surgery or the ispilateral arm, present at least 4 days a week and with an intensity of 3 or more on a numeric rating scale from 0 to 10 (0=no pain and 10=worst possible pain).

## Results

**Figure 1:**
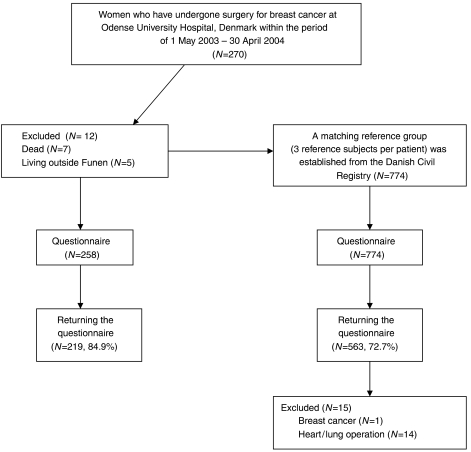
Inclusion and exclusion of women with and without breast cancer in the study.

Questionnaires were mailed to 258 breast cancer patients and 774 reference subjects. After one written reminder, 219 (84.9%) of the breast cancer patients and 563 (72.7%) of the reference subjects had returned the questionnaire (Figure 1). The percentages of patients and controls who needed reminders were 16.4 and 15.8%, respectively. The prevalence of PMPS in the group of breast cancer patients was 23.9% and the prevalence of PMPS-like symptoms in the reference group was 10.0%. The odds ratio for developing PMPS after surgery for breast cancer was 2.88 (95% CI 1.84–4.51).

### Demographic characteristics

There was no significant difference in age between the responders of the breast cancer patients and the reference subjects (median age being 61.1 (IQR 54.2–68.1) *vs* 60.3 (IQR 53.7–66.7). In the reference group, the median age was younger for subjects who reported pain similar to PMPS (median age being 55.9 (IQR 49.7–61.8) *vs* 60.5 (IQR 54.1–66.8) (*P*=0.003). For other demographic characteristics, there were a higher proportion of subjects from the reference group that had children and twice as many had a family history of chronic pain conditions (Table 1). The characteristics are shown in Table 1. *Post hoc* analysis showed that the odds ratio for developing PMPS increased to 3.7 (95% CI 1.82–8.30) when adjusting for family history. Positive family history increased the risk of PMPS-like symptoms in both groups. However, as controls were more likely to have a positive family history, this reduces the difference in PMPS-like symptoms between cases and controls.

### Risk factors

The results of both univariate and multiple logistic regression analysis are shown in Table 2. When using multiple regression analysis, three significant risk factors were identified: Having undergone breast surgery earlier (OR 8.12), tumour located in the upper lateral quarter (OR 6.48) and young age (OR 1.04). Not-associated risk factors included the following: chemotherapy, axillary dissection, mastectomy, smoking, tumour size and radiation therapy.

### The location and character of pain

The distribution of the location of pain and the character of pain in the group of breast cancer patients with PMPS and in the reference group is shown in Table 1. The majority of the breast cancer patients with PMPS had pain located in the axilla/arm (80.8%) and in the area of the scar (55.8%), and 75.0% of the patients had pain in more than one location. These findings were significantly higher than in the reference group. There were no significant differences with respect to the character of pain.

### The type of breast surgery, axillary intervention and location of pain

The location of pain subdivided according to the type of breast surgery and axillary intervention is shown in Table 3. When applying logistic regression analysis, we found that the odds ratio for developing pain in the mamma was 0.27 (95% CI 0.11–0.72) for lumpectomy, 2.91 (95% CI 0.59–14.4) for sentinel node biopsy and 1.99 (95% CI 0.43–9.20) for axillary dissection. The odds ratio for developing pain in the shoulder, axilla and arm was 0.76 (95% CI 0.36–1.58) for lumpectomy, 0.88 (95% CI 0.25–3.12) for sentinel node biopsy and 1.84 (95% CI 0.58–5.83) for axillary dissection.

Taking mastectomy and lumpectomy together, the frequency of pain in general and at any location increased with the extent of axillary intervention (Table 4).

### Radiation therapy and location of pain

In Table 5, the relation between radiation therapy and the location of pain is shown. A higher frequency of pain was observed in the three categories when the radiation therapy included the supraclavicular fields and the axillary region.

### Systemic therapy

None of the patients received treatment with taxanes or aromatase inhibitors.

### Pain treatment

The frequency of consumption of analgesics did not differ significantly between the two groups. Among the breast cancer patients with PMPS, 38.5% reported use of analgesics compared with 52.8% of the reference subjects with pain. The frequency of analgesic consumption was compared between the two groups, the options being ‘Every day’, ‘every other day’, ‘once a week’, ‘every other week’ and ‘once a month’. The distribution of answers in the strata did not differ significantly, *P*=0.349.

### The impact of pain on daily life

**Figure 2:**
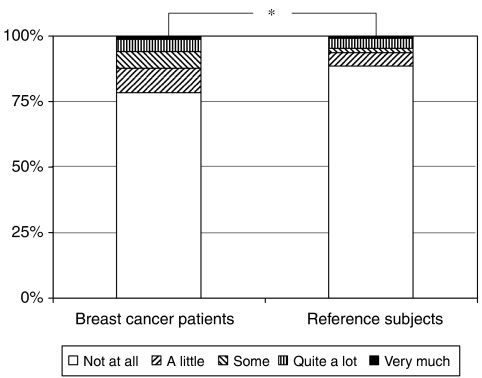
The impact of pain on daily life. ^*^Significant difference (*P*=0.0004).

More breast cancer patients than reference subjects reported that pain interfered with daily life ‘really much’ (1.4 *vs* 0.8%), ‘quite a lot’ (4.6 *vs* 4.0%), ‘some’ (6.4 *vs* 1.5%) and ‘a little bit’ (9.2 *vs* 5.0%). The distribution of answers in the strata differed between the two groups (*z*=3.52, *P*=0.0004) (Figure 2).

When looking at the patients with pain, who reported pain interfering ‘really much’ or ‘quite a lot’ (*n*=13), a mean pain score of 7.2 (range 3–10) on a numeric rating scale from 0–10 was found. All 13 patients had pain in the shoulder/axilla/arm and 6 patients had, in addition to this, pain in the mamma. No other common features were found regarding the pain symptoms for this subgroup.

## Discussion

To our knowledge, this is the first population-based study of chronic pain after surgery for breast cancer that includes a comparable reference group.

The prevalence of PMPS in this study was found to be 24% as compared with PMPS-like symptoms in 10% of the reference group. The odds ratio for developing PMPS after treatment for breast cancer was 2.9. This prevalence for PMPS in women after surgery for breast cancer is similar to results obtained by Smith *et al* in (1999) (29%) and Carpenter *et al* in (1998) (27%).

In one study, the prevalence has been found to be higher in low volume units than in high volume units (Tasmuth *et al*, 1999). The prevalence of PMPS-like symptoms in women who had not had surgery for breast cancer is surprisingly high and mandates a conservative view on the raw prevalence figures.

The surgical procedures used in the region in the period 2003–2008 have remained principally unchanged. Mammographic screening had been introduced 10 years earlier and thus resulted in an increase in the number of women who could have breast-conserving procedures. The sentinel node technique became more widely used in the last part of the period and this has led to an increase in axillary-sparing procedures.

Three risk factors for developing PMPS were identified in this study: having undergone breast surgery earlier, tumour located in the upper lateral quarter and young age. Earlier surgery in the same breast appears to be a logical risk factor for PMPS. Regarding the location of the tumour, previous studies have not evaluated this variable as a risk factor. With tumours in close relation to the axilla, there is a higher risk of damaging nerves in the area that may increase the risk of subsequent chronic pain. Young age has also been reported to be a predisposing factor to PMPS in other studies (Tasmuth *et al*, 1995; Smith *et al*, 1999; Poleshuck *et al*, 2006). It has previously been suggested that this may be caused by the more aggressive character of disease in this group of patients, requiring more invasive surgical procedures and chemotherapy (Kroman *et al*, 2000; Colleoni *et al*, 2002). However, in this study, these factors are included in the multiple regression analysis, and this indicates that other factors may account for the fact that PMPS is seen more often among young patients.

Two possible risk factors, which have been considered previously, are the sectioning of the intercostobrachial nerve (Abdullah *et al*, 1998; Torresan *et al*, 2003) and axillary dissection (Vecht, 1990; Maunsell *et al*, 1993; Hack *et al*, 1999; Kakuda *et al*, 1999; Johansen *et al*, 2000). However, one study has shown the opposite result, with postmastectomy pain occurring without damage to the intercostobrachial nerve and in women without axillary dissection (Carpenter *et al*, 1999). In this study, it was not possible to obtain information about the sectioning or preservation of the intercostobrachial nerve among the patients included. Axillary dissection came out as a risk factor in univariate analysis, but this effect could not be reproduced in the multivariate analysis.

We did not find any significant difference in the description of pain between breast cancer patients and the reference group. However, the location of the pain did differ between the two groups, with the majority of the breast cancer patients having pain in the shoulder, in the area of the scar and in more than one location. These findings are in agreement with earlier findings (Stevens *et al*, 1995; Carpenter *et al*, 1998). When using logistic regression analysis, we found that pain located in the mamma was seen approximately four times less frequent in the group of women who had undergone lumpectomy compared with the women who had undergone mastectomy. Sentinel node biopsy and axillary dissection was not associated with a significant effect on this pain location. Neither the type of breast surgery nor the type of axillary intervention had a significant impact on the frequency of pain located in the mamma. A similar pattern was observed for radiation therapy. An increase in pain located in the breast and especially pain located in the shoulder and arm was seen, when the radiation field included supraclavicular glands and the axillary region. A recent study, including 278 breast cancer patients, has addressed the impact of different types of pain on the degree of disability and distress (Kudel *et al*, 2007). Three types of pain were studied: phantom breast pain, scar pain and other mastectomy-related pain. In this study, ‘other mastectomy-related pain’ was found to be the strongest predictor of disability and distress. Demographic and surgical factors were not consistent predictors of pain or function.

For this type of study, some methodological limitations must be considered. The study group consisted of women who had undergone treatment for breast cancer at one centre, a teaching hospital, and all the women in the study group were Caucasian, that is, we do not know if the figures can be generalised to other settings. Further, it is possible that the percentage of women who did not have PMPS or PMPS-like symptoms was higher in the group of non-responders than in the group of responders. As the response rates in the study group and in the control group were 85 and 73%, respectively, the prevalence of PMPS and the odds ratio for developing PMPS may have been overestimated. There are no specific questionnaires for identifying and evaluating pain after surgery in breast cancer patients, and the questionnaires developed for estimating neuropathic pain components (Bennett, 2001; Krause and Backonja, 2003; Bouhassira *et al*, 2005; Freynhagen *et al*, 2006; Portenoy, 2006) were considered not to be suited for the present purpose. Thus, we chose to make a questionnaire specifically for this study and we have no data to support its validity. Women with possible reoccurrence of cancer were included in the study and this is a potential bias of the results. The tumour itself may have caused pain and may thus have caused a higher prevalence of pain in the breast cancer group. Finally, our study comprised 219 patients, and the analysis of the risk factors may have been hampered by the low number of patients.

Chronic pain after surgery has been reported to develop in 5–60% of patients after operations such as thoracotomy, hip arthroplasty, hysterectomy, thoracotomy and inguinal hernia repair (Perttunen *et al*, 1999; Aasvang and Kehlet, 2005; Nikolajsen *et al*, 2006; Maguire *et al*, 2006; Pluijms *et al*, 2006; Kehlet *et al*, 2006; Brandsborg *et al*, 2007). For hysterectomy, it was found that about 14% had pelvic pain more than 2 days a week and risk factors were preoperative pelvic pain, pain as the main indicator for surgery and pain problems elsewhere (Brandsborg *et al*, 2007). Pain after total hip arthroplasty, which limited daily living to a moderate to very severe degree, occurred with a frequency of about 12%, and the risk factors were intensity of early postoperative pain and pain complaints from other areas of the body (Nikolajsen *et al*, 2006). Prevalence of post-thoracotomy pain was observed to be 21%, when evaluated 6–7 years after surgery, and risk factors were age, consultant and time since surgery (Maguire *et al*, 2006).

Only 22% of the breast cancer patients reported that the pain had an impact on the daily life and use of analgesics was low. These findings suggest that the severity of PMPS, in general, is moderate, which is in agreement with earlier studies (de Vries *et al*, 1994). The majority of the breast cancer patients with severe pain have pain located in the shoulder, axilla or arm. This adds evidence to the finding of tumour located in the upper lateral quarter being an important risk factor, as operation in this area may tend to cause more nerve damage than surgery in other areas of the breast. This finding has not been reported in earlier studies.

In conclusion, it seems that, although recent advances in the diagnostic and surgical procedures have reduced the frequency of the more invasive surgical procedures, there is still a considerable risk of developing PMPS after treatment for breast cancer, and development of preventive measures as well as treatments of the syndrome are highly relevant.

## Figures and Tables

**Table 1:** Demographics of all subjects included in the study and location and character of pain in subjects from breast cancer group (52/219) and reference group (48/563) with PMPS

	**Breast cancer patients**	**Reference subjects**	***P*-value**
*Demographics* (%)			
Married	65	66	0.78
Children*	88	94	0.04
Working	36	43	0.08
Smoking	26	26	0.91
Alcohol abuse	7	7	0.78
Family history of chronic pain conditions*	9	18	<0.01
			
*Location of pain* (%)			
Scar	56	0	
Chest wall	35	21	0.14
Shoulder*	40	73	<0.01
Axilla/arm*	81	48	<0.01
Other	21	27	0.50
>1 location*	75	48	<0.01
			
*Character of pain* (%)			
Throbbing	42	48	0.52
Shooting	50	40	0.27
Burning	23	13	0.16
Oppressing	37	29	0.55

* Significant difference: *P*<0.05.

**Table 2:** Possible risk factors for developing the postmastectomy pain syndrome (PMPS)

	**Univariate analysis**		**Multivariate models**	
	**Logistic regression**		**Logistic regression**	
**Variable**	**Odds ratio of developing PMPS (95% CI)**	***P*-value**	**Odds ratio of developing PMPS (95% CI)**	***P*-value**
Chemotherapy (CMF/CEF)	2.78 (1.29–6.02)^*^	0.01	1.63 (0.64–4.17)	0.31
Tumour located in the upper lateral quarter	2.78 (1.22–6.34)^*^	0.02	6.48 (2.24–18.77)^*^	0.00
Axillary dissection	2.03 (1.06–3.89)^*^	0.03	1.37 (0.58–3.19)	0.47
Young age	1.03 (1.01–1.05)^*^	0.03	1.04 (1.00–1.08)^*^	0.04
Mastectomy (*vs* lumpectomy)	2.01 (1.07–3.79)^*^	0.03	0.81 (0.25–2.69)	0.74
Smoking	2.03 (1.03–4.00)^*^	0.04	1.72 (0.75–3.96)	0.20
Having undergone breast surgery earlier	2.20 (0.99–4.90)	0.05	8.12 (2.39–27.64)^*^	0.001
Tumour size	1.02 (1.00–1.05)	0.05	1.02 (0.99–1.06)	0.18
Metastasis to the local lymph nodes	1.73 (0.80–3.39)	0.11		
Positive oestrogen receptorstatus	0.55 (0.24–1.29)	0.17		
Endocrine therapy	1.47 (0.68–3.20)	0.33		
Radiation therapy	0.75 (0.40–1.42)	0.38	0.56 (0.20–1.56)	0.27
Grade of malignancy	1.18 (0.74–1.92)	0.48		
Body mass index	1.00 (0.90–1.11)	0.95		

List of possible risk factors determined by odds ratio. ^*^Significant risk factor (*P*<0.05).

**Table 3:** Type of breast surgery and location of pain

**Axillary intervention**	**Mamma *n* (%)**	**Shoulder, axilla and arm *n* (%)**	**Any pain *n* (%)**
*Lumpectomy*			
No axillary intervention (*n*=11)	0 (0)	1 (9.1)	1 (9.1)
Sentinel node (*n*=64)	5 (7.8)	7 (10.9)	8 (12.5)
Axillary dissection (*n*=46)	5 (10.9)	8 (28.3)	8 (28.3)
			
*Mastectomy*			
No axillary intervention (*n*=13)	2 (15.4)	3 (23.1)	3 (23.1)
Sentinel node (*n*=16)	6 (37.5)	4 (25.0)	6 (37.5)
Axillary dissection (*n*=69)	13 (19.1)	18 (26.5)	20 (29.4)

**Table 4:** Axillary intervention and location of pain

**Axillary intervention**	**Mamma, % (95% CI)**	**Shoulder, axilla and arm, % (95% CI)**	**Any pain, % (95% CI)**
No axillary intervention (*n*=24)	8.3 (1.0–27.0)	16.7 (4.7–37.4)	16.7 (4.7–37.4)
Sentinel node (*n*=80)	13.8 (7.1–23.3)	13.8 (7.1–23.3)	17.5 (9.9–27.6)
Axillary dissection (*n*=115)	16.5 (10.3–46.0)	27.8 (19.9–37.0)	29.6 (21.4–88.0)

**Table 5:** Radiation therapy and location of pain

**Radiation therapy**	**Mamma, % (95% CI)**	**Shoulder, axilla and arm, % (95% CI)**	**Any pain, % (95% CI)**
No radiation therapy (*n*=83)	8.3 (1.0–27.0)	16.7 (4.7–37.4)	16.7 (4.7–37.4)
Breast and clavicle (*n*=91)	8.8 (7.0–21.9)	13.8 (7.1–23.3)	17.5 (9.9–27.6)
Breast and clavicle+medial axillary region (*n*=32)	16.5 (10.3–4.6)	27.8 (19.9–37.0)	29.6 (21.4–8.8)
Breast and clavicle+ all the axillary region (*n*=13)	30.8 (9.1–61.4)	53.8 (25.1–80.8)	53.8 (25.1–80.8)

## References

[bib1] Aasvang E, Kehlet H (2005) Chronic postoperative pain: the case of inguinal herniorrhaphy. Br J Anaesth 95(1): 69–76 15531621 10.1093/bja/aei019

[bib2] Abdullah TI, Iddon J, Barr L, Baildam AD, Bundred NJ (1998) Prospective randomized controlled trial of preservation of the intercostobrachial nerve during axillary node clearance for breast cancer. Br J Surg 85: 1443–1445 9782034 10.1046/j.1365-2168.1998.00843.x

[bib3] Bennett M (2001) The LANSS Pain Scale: the Leeds assessment of neuropathic symptoms and signs. Pain 92(1–2): 147–157 11323136 10.1016/s0304-3959(00)00482-6

[bib4] Bouhassira D, Attal N, Alchaar H, Boureau F, Brochet B, Bruxelle J, Cunin G, Fermanian J, Ginies P, Grun-Overdyking A, Jafari-Schluep H, Lantéri-Minet M, Laurent B, Mick G, Serrie A, Valade D, Vicaut E (2005) Comparison of pain syndromes associated with nervous or somatic lesions and development of a new diagnostic questionnaire (DN4). Pain 114(1–2): 29–36 15733628 10.1016/j.pain.2004.12.010

[bib5] Brandsborg B, Nikolajsen L, Hansen CT, Kehlet H, Jensen TS (2007) Risk factors for chronic pain after hysterectomy. Anesthesiology 106: 1003–1112 17457133 10.1097/01.anes.0000265161.39932.e8

[bib6] Carpenter JS, Andrykowski MA, Sloan P, Cunningham L, Cordova MJ, Studts JL, McGrath PC, Sloan D, Kenady DE (1998) Postmastectomy/postlumpectomy pain in breast cancer survivors. J Clin Epidemiol 51: 1285–1292 10086821 10.1016/s0895-4356(98)00121-8

[bib7] Carpenter JS, Sloan B, Andrykowski MA, McGrath P, Sloan D, Rexford T, Kenady D (1999) Risk factors for pain after mastectomy/lumpectomy. Cancer Practice 7(2): 66–70 10352063 10.1046/j.1523-5394.1999.07208.x

[bib8] Colleoni M, Rotmensz N, Robertson C, Orlando L, Viale G, Renne G, Luini A, Veronesi P, Intra M, Orecchia R, Catalano G, Galimberti V, Nolé F, Martinelli G, Goldhirsch A (2002) Very young women (<35 years) with operable breast cancer: features of disease after presentation. Ann Oncol 13: 273–279 10.1093/annonc/mdf039 11886005

[bib9] De Vries JE, Timmer PR, Erftemeier EJ, van der Weele LT (1994) Breast pain after breast concerving therapy. Breast 3: 151–154

[bib10] Freynhagen R, Baron R, Gockel U, Tölle T (2006) painDETECT: a new screening questionnaire to detect neuropathic components in patients with back pain. Curr Med Res Opin 22: 1911–1920 17022849 10.1185/030079906X132488

[bib11] Hack TF, Cohen L, Katz J, Robson LS, Goss P (1999) Physical and psychological morbidity after axillary lymph node dissection for breast cancer. J Clin Oncol 17(1): 143–149 10458227 10.1200/JCO.1999.17.1.143

[bib12] Ivens D, Hoe AL, Podd TJ, Hamilton CR, Taylor I, Royle GT (1992) Assessment of morbidity from complete axillary dissection. Br J Cancer 66: 136–138 1637663 10.1038/bjc.1992.230 PMC1977908

[bib13] Johansen J, Overgaard J, Blichert-Toft M, Overgaard M (2000) Treatment morbidity associated with the management of the axilla in breast-conserving therapy. Acta Oncologica 39(3): 349–354 10987232 10.1080/028418600750013122

[bib14] Jung BF, Ahrendt GM, Oaklander AL, Dworkin RH (2003) Neuropathic pain following breast cancer surgery: proposed classification and research update. Pain 104: 1–13 12855309 10.1016/s0304-3959(03)00241-0

[bib15] Kakuda JT, Stuntz M, Trivedi V, Klein SR, Vargas HI (1999) Objective assessment of axillary morbidity in breast cancer treatment. Am Surg 65: 995–998 10515551

[bib16] Kehlet H, Jensen TS, Woolf CJ (2006) Persistent postsurgical pain: risk factors and prevention. Lancet 367: 1618–1625 16698416 10.1016/S0140-6736(06)68700-X

[bib17] Krause SJ, Backonja MM (2003) Development of a neuropathic pain questionnaire. Clin J Pain 19(5): 306–314 12966256 10.1097/00002508-200309000-00004

[bib18] Kretschmer T, Nguyen DH, Beuerman RW, Happel LT, England JD, Tiel RL, Kline DG (2002) Painful neuromas: a potential role for a structural transmembrane protein, ankyrin G. J Neurosurg 97: 1424–1431 12507143 10.3171/jns.2002.97.6.1424

[bib19] Kroman N, Jensen M, Wohlfahrt J, Mouridsen HT, Andersen PK, Melbye M (2000) Factors influencing the effect of age on prognosis in breast cancer: population based study. BMJ 320: 474–479 10678859 10.1136/bmj.320.7233.474 PMC27289

[bib20] Kudel I, Edwards RR, Kozachik S, Block BM, Agarwal S, Heinberg LJ, Haythornthwaite J, Raja SN (2007) Predictors and consequences of multiple persistent postmastectomy pains. J Pain Symptom Manage 34(6): 619–627 17629668 10.1016/j.jpainsymman.2007.01.013

[bib21] Kuehn T, Klauss W, Darsow M, Regele S, Flock F, Maiterth C, Dahlbender R, Wendt I, Kreienberg R (2000) Long-term morbidity following axillary dissection in breast cancer patients – clinical, assessment, significance for life quality and the impact of demographic, oncologic and therapeutic factors. Breast Cancer Res Treat 64: 275–286 11200778 10.1023/a:1026564723698

[bib22] Macdonald L, Bruce J, Scott NW, Smith WCS, Chambers WA (2005) Long-term follow-up of breast cancer survivors with post-mastectomy pain syndrome. Br J Cancer 92: 225–230 15655557 10.1038/sj.bjc.6602304 PMC2361843

[bib23] Macrae WA (2001) Chronic pain after surgery. Br J Anaesth 87: 88–98 11460816 10.1093/bja/87.1.88

[bib24] Maguire MF, Ravenscroft A, Beggs D, Dufy JP (2006) A questionnaire study investigating the prevalence of the neuropathic component of chronic pain after thoracic surgery. Eur J Cardiothorac Surg 29(5): 800–805 16581259 10.1016/j.ejcts.2006.02.002

[bib25] Mansel RE, Fallowfield L, Kissin M, Goyal A, Newcombe RG, Dixon JM, Yiangou C, Horgan K, Bundred N, Monypenny I, England D, Sibbering M, Abdullah TI, Barr L, Chetty U, Sinnett DH, Fleissig A, Clarke D, Ell PJ (2006) Randomized multicenter trial of sentinel node biopsy *vs* standard axillary treatment in operable breast cancer: the ALMANAC Trial. J Natl Cancer Inst 98(9): 599–609 16670385 10.1093/jnci/djj158

[bib26] Maunsell E, Brisson J, Deschenes L (1993) Arm problems and psychological distress after surgery for breast cancer. CJS 36(4): 315–320 8370012

[bib27] Miguel R, Kuhn AM, Shons AR, Dyches P, Ebert MD, Peltz ES, Nguyen K, Cox CE (2001) The effect of sentinel node selective axillary lymphadenectomy on the incidence of postmastectomy pain syndrome. Cancer Control 8(5): 427–430 11579339 10.1177/107327480100800506

[bib28] Nikolajsen L, Brandsborg B, Lucht U, Jensen TS, Jensen TS (2006) Chronic pain following total hip arthroplasty: a nationwide questionnaire study. Acta Anaesthesiol Scand 50: 495–500 16548863 10.1111/j.1399-6576.2006.00976.x

[bib29] Peintinger F, Reitsamer R, Stranzl H, Ralph G (2003) Comparison of quality of life and arm complaints after axillary lymph node dissection *vs* sentinel node biopsy in breast cancer patients. Br J Cancer 89: 648–652 12915872 10.1038/sj.bjc.6601150 PMC2376906

[bib30] Perkins FM, Kehlet H (2000) Chronic pain as an outcome of surgery: a review of predictive factors. Anesthesiology 93: 1123–1133 11020770 10.1097/00000542-200010000-00038

[bib31] Perttunen K, Tasmuth T, Kalso E (1999) Chronic pain after thoracic surgery: a follow-up study. Acta Anaesthesiol Scand 43(5): 563–567 10342006 10.1034/j.1399-6576.1999.430513.x

[bib32] Pluijms WA, Steegers MA, Verhagen AF, Scheffer GJ, Wilder-Smith OH (2006) Chronic postthoracatomy pain: a retrospective study. Acta Anaestesiol Scand 50: 804–808 10.1111/j.1399-6576.2006.01065.x 16879462

[bib33] Poleshuck EL, Katz J, Andrus CH, Hogan LA, Jung BF, Kulick DI, Dworkin RH (2006) Risk factors for chronic pain following breast cancer surgery: a prospective study. J Pain 7(9): 626–634 16942948 10.1016/j.jpain.2006.02.007 PMC6983301

[bib34] Portenoy R (2006) Development and testing of a neuropathic pain screening questionnaire: ID Pain. Curr Med Res Opin 22(8): 1555–1565 16870080 10.1185/030079906X115702

[bib35] Rosso R, Scelsi M, Carnevali L (2000) Granular cell neuroma. Arch Pathol Lab Med 124: 709–711 10782152 10.5858/2000-124-0709-GCTN

[bib36] Schijven MP, Vingerhoets AJ, Rutten HJ, Nieuwenhuijzen GA, Roumen RM, van Bussel ME, Voogd AC (2003) Comparison of morbidity between axillary lymph node dissection and sentinel node biopsy. Eur J Surg Oncol 29(4): 341–350 12711287 10.1053/ejso.2002.1385

[bib37] Shons AR, Cox CE (2001) Breast cancer: advances in surgical management. Plast Reconstr Surg 107(2): 541–549 11214073 10.1097/00006534-200102000-00035

[bib38] Smith WCS, Bourne D, Squair J, Phillips DP, Chambers WA (1999) A retrospective cohort study of post mastectomy pain syndrome. Pain 83: 91–95 10506676 10.1016/s0304-3959(99)00076-7

[bib39] Stevens PE, Dibble SL, Miaskowski C (1995) Prevalence, characteristics, and impact of postmastectomy pain syndrome: an investigation of women's experiences. Pain 61(1): 61–68 7644250 10.1016/0304-3959(94)00162-8

[bib40] Tasmuth T, Blomqvist C, Kalso E (1999) Chronic post-treatment symptoms in patients with breast cancer operated in different surgical units. Eur Journal Surg Oncol 25: 38–43 10188853 10.1053/ejso.1998.0597

[bib41] Tasmuth T, Kataja M, Blomquist C, von Smitten K, Kalso E (1997) Treatment-related factors predisposing to chronic pain in patients with breast cancer. Acta Oncologica 36(6): 625–630 9408154 10.3109/02841869709001326

[bib42] Tasmuth T, von Smitten K, Hietanen P, Mataja M, Kalso E (1995) Pain and other symptoms after different treatment modalities of breast cancer. Annals Oncol 6: 453–459 10.1093/oxfordjournals.annonc.a059215 7669710

[bib43] Tasmuth T, von Smitten K, Kalso E (1996) Pain and other symptoms during the first year after radical and conservative surgery for breast cancer. Br J Cancer 74: 2024–2031 8980408 10.1038/bjc.1996.671 PMC2074824

[bib44] Torresan RZ, Cabello C, Conde DM, Brenelli HB (2003) Impact of the preservation of the intercostobrachial nerve in axillary lymphadenectomy due to breast cancer. The Breast J 9(5): 389–392 12968959 10.1046/j.1524-4741.2003.09505.x

[bib45] Vecht CJ (1990) Arm pain in the patient with breast cancer. J Pain and Symptom Manage 5(2): 109–117 2348086 10.1016/s0885-3924(05)80024-7

[bib46] Wallace MS, Wallace AM, Lee J, Dobke MK (1996) Pain after breast surgery: a survey of 282 women. Pain 66: 195–205 8880841 10.1016/0304-3959(96)03064-3

[bib47] Wong L (2001) Intercostal neuromas: a treatable cause of postoperative breast surgery pain. Ann Plast Surg 46: 481–484 11352419 10.1097/00000637-200105000-00004

